# The effect of Ba Duan Jin exercise intervention on cardiovascular disease: a meta-analysis of randomized controlled trials

**DOI:** 10.3389/fpubh.2024.1425843

**Published:** 2024-08-06

**Authors:** Jiali Chen, Man Zhang, Yihao Wang, Ziyu Zhang, Shuyan Gao, Yafei Zhang

**Affiliations:** ^1^Global Medical Research Promotion, Graduate School of Medicine Science and Technology, Shinshu University, Matsumoto, Japan; ^2^Department of Physical Education, Hebei Medical University, Shijiazhuang, China; ^3^Nursing School, Graduate School, Hebei Medical University, Shijiazhuang, China; ^4^Department of Physical Education, Clinical College, Hebei Medical University, Shijiazhuang, China

**Keywords:** Ba Duan Jin, effect, cardiovascular diseases, systematic review, meta-analysis

## Abstract

**Background:**

There is a growing interest in the use of complementary therapies for the prevention of disease and the maintenance of health. Furthermore, complementary therapies that incorporate exercise are becoming increasingly prevalent among the older adult, and thus may represent a crucial strategy for the primary and secondary prevention of cardiovascular disease (CVD). Exercise therapy, as a means to prevent and treat cardiovascular diseases, has been gradually applied in clinical practice. It has the advantages of reducing mortality, improving clinical symptoms, restoring physical function and improving quality of life. In recent years, traditional Chinese sports such as Ba Duan Jin and Qigong have developed rapidly. Therefore, a comprehensive systematic review is required to examine interventions involving Ba Duan Jin exercise in healthy adults or those at increased risk of CVD in order to determine the effectiveness of Ba Duan Jin exercise for the primary prevention of CVD.

**Objective:**

To investigate the effect of Ba Duan Jin exercise intervention for the primary prevention of cardiovascular diseases.

**Methods:**

Eight databases were systematically searched from inception to July, 2024 for randomized controlled trials (RCTs) to evaluated the impact of Ba Duan Jin exercise intervention on cardiovascular diseases. The search terms were “Cardiovascular diseases” “Ba Duan Jin” and “Randomized controlled.” The Cochrane risk assessment tool was used to evaluate the study quality, and the meta-analysis was performed using Rev. Man 5.4 software.

**Results:**

Seventeen completed trials were conducted with 1,755 participants who were randomly assigned and met the inclusion criteria. All 17 studies were conducted in China. The meta-analysis indicates that Ba Duan Jin exercise therapy can provide long-term benefits (20–30 years) by reducing all-cause mortality (*RR* = 0.55, 95% *CI*: 0.44–0.68, *p* < 0.01) and stroke mortality (*RR* = 0.49, 95% *CI*: 0.36–0.66, *p* < 0.01) in hypertensive patients. Subgroup analyses reveal that Ba Duan Jin exercise therapy decreases SBP (*MD* = −4.05, 95% *CI* = −6.84 to −1.26, *p* < 0.01) and DBP (*MD* = −3.21, 95% *CI* = −5.22 to −1.20, *p* < 0.01) levels in patients with essential hypertension, significantly reduces serum TC (*MD* = −0.78, 95% *CI* = −1.06 to −0.50, *p* < 0.01), TG (*MD* = −0.78, 95% *CI* = −0.93 to −0.62, *p* < 0.01), and LDL-C (*MD* = −0.76, 95% *CI* = −0.92 to −0.60, *p* < 0.01) levels in patients with hyperlipidemia, increases HDL-C (*MD* = 0.32, 95% *CI* = 0.14–0.51, *p* < 0.01) levels, and produces beneficial effects on cardiovascular function. Additionally, it can alleviate anxiety (*MD* = −3.37, 95% *CI* = −3.84 to −2.89, *p* < 0.01) and improve sleep quality (*MD* = −2.68, 95% *CI* = −3.63to −1.73, *p* < 0.01).

**Conclusion:**

Ba Duan Jin exercise therapy can improve the physical and mental condition and quality of life of patients with cardiovascular diseases, and it is worthy of further promotion and application in clinical practice.

**Systematic review registration:**

PROSPERO, identifier: https://www.crd.york.ac.uk/prospero/display_record.php?ID=CRD42024496934.

## 1 Introduction

Cardiovascular diseases (CVDs) continue to be the primary cause of global deaths and disabilities. Over the past decade, the global number of deaths from CVD has increased by 12.5%, attributing to approximately one third of all deaths worldwide (1). The development of CVD is influenced by various factors including changes in health-related behaviors such as tobacco use, unhealthy diet, physical inactivity, alcohol consumption and high levels of stress. Each of these factors plays a significant role in the development and progression of CVD (2–4). Between 1990 and 2019, there was a significant increase in the number of deaths due to cardiovascular disease (CVD), rising from 12.1 million in 1990 to 18.6 million in 2019 (5). As a result, there is a growing need for public health interventions aimed at addressing the prevalence of CVD, and promoting healthier lifestyle choices to mitigate their impact.

Traditional Chinese exercise, such as Tai Chi and Qigong, has been extensively utilized in the management and treatment of various diseases. These exercises have shown positive effects in conditions like primary hypertension and cancer (6, 7). There is a widespread pandemic of physical inactivity that seems to mirror the high prevalence of cardiovascular disease (CVD). However, regular physical activity (PA) and exercise have a significant impact not only on primary cardiovascular prevention but also on secondary prevention (8–10). The Ba Duan Jin, also known as the Eight Section Brocade, is a traditional Chinese exercise that has been practiced for thousands of years. It is highly regarded as a valuable component of Chinese culture and is renowned for its health benefits (11). The practice of Ba Duan Jin is known to have positive effects on the mental, emotional, and social well-being of individuals (12). As a form of qigong, Ba Duan Jin is believed to facilitate the flow of qi, or life energy, throughout the body, which in turn contributes to improved health and vitality (13).

Ba Duan Jin has demonstrated promising results in enhancing various physical and neurological functions in patients. Research studies have indicated its potential to improve balance ability (14), motor skills, trunk stability (15), neurological functions (16), ability to perform daily living activities (17), and overall quality of life (18, 19). Additionally, it has been associated with reducing symptoms of anxiety and depression (20, 21). These findings suggest that Ba Duan Jin could be a valuable addition to rehabilitation and wellness programs for individuals seeking to improve their physical and mental well-being.

To date, there have been few randomized controlled trials (RCTs) investigating the effectiveness of Ba Duan Jin exercise for the prevention of cardiovascular disease (CVD), and even fewer systematic reviews exploring this topic. Three systematic reviews of interest examined the efficacy of Ba Duan Jin exercise for hypertension. The findings indicated that Ba Duan Jin exercise was associated with a reduction in blood pressure in individuals with hypertension (22). These findings are consistent with the findings of Bugni et al., which indicated that internal qigong was effective in decreasing blood pressure among those with essential hypertension when compared with no-treatment controls (23). However, it was not as effective as drug controls or conventional exercise controls. A further systematic review was conducted to assess the efficacy of Ba Duan Jin exercise in the management of essential hypertension (24). In comparison to control interventions, Ba Duan Jin exercise appears to be an efficacious physical exercise modality for the treatment of essential hypertension. The duration of the training period can influence the observed effects. Furthermore, a meta-analysis was conducted to investigate the impact of traditional Chinese exercises, namely Qigong and Tai Chi, on quality of life in individuals with essential hypertension. This systematic review and meta-analysis of randomized controlled trials suggests that Tai Chi may be an effective therapeutic approach to enhance quality of life in patients with essential hypertension (6).

It is notable that none of the above systematic reviews investigated the efficacy of Ba Duan Jin exercise therapy in the prevention of cardiovascular disease (6, 22–24). The main objective of this systematic review and meta-analysis is to investigate the efficacy of pure Ba Duan Jin exercise in the prevention of cardiovascular diseases, with the aim of providing new insights into the prevention of the cardiovascular system and managing cardiovascular health.

## 2 Methods

The study was conducted following the guidelines of the Preferred Reporting Items for Systematic Reviews and Meta-Analyses (PRISMA) and was also registered on PROSPER (registration number: CRD42024496934).

### 2.1 Search strategy

The researchers conducted separate searches for English and Simplified Chinese studies related to the association between Ba Duan Jin and cardiovascular disease (CVD). They employed keywords such as Ba Duan Jin, Eight trigrams boxing, Cardiovascular Disease, and CVD to locate pertinent studies in various databases including PubMed, Cochrane Library, Embase, Web of Science, Chinese National Knowledge Infrastructure (CNKI), Wan Fang Data, Chinese Science and Technology Journal Full-Text Database (VIP), and Chinese Biomedical Literature Database (CBM) to collect randomized controlled trials examining the impacts of Ba Duan Jin on cardiovascular disease, until July, 2024. Additionally, the reference lists of relevant publications were manually examined to uncover further studies. A detailed search strategy for PubMed was shown in Supplementary Table S1.

### 2.2 Criteria for inclusion

#### 2.2.1 Types of studies

Randomized controlled trials (RCT).

#### 2.2.2 Types of participant

Adults aged 18 years and older from the general population, as well as adults at high risk of cardiovascular disease (CVD), were included in the study.

#### 2.2.3 Types of interventions

The experimental group received a single traditional Chinese exercise, such as Ba Duan Jin or qigong.

In order to avoid confounding, this review did not include multi-factorial lifestyle intervention trials. The control interventions could be routine care or no intervention.

#### 2.2.4 Types of outcome measures

##### 2.2.4.1 Primary outcomes

All-cause mortality, stroke mortality, myocardial infarction mortality.

##### 2.2.4.2 Secondary outcomes

Changes in blood pressure (systolic and diastolic blood pressure) and blood lipids [total cholesterol (TC), high-density lipid cholesterol (HDL-C), low-density lipid cholesterol (LDL-C)], triglycerides (TG), anxiety and sleep quality.

### 2.3 Criteria for exclusion

The study exclusion criteria were applied as follows: (1) Individuals with a history of myocardial infarction (MI), stroke, revascularization procedures (such as coronary artery bypass grafting or percutaneous transluminal coronary angioplasty), people with angina, or those with angiographically defined coronary heart disease (CHD). (2) The intervention did not match or combined with other interventions. (3) Studies published in books or conference proceedings. (4) Unavailability of the complete study.

### 2.4 Study selection

The search results were imported into Endnote software. Studies were independently screened by two researchers using the above inclusion criteria. Any differences between the two researchers are resolved through discussion with a third researcher. After culling duplicate studies, two researchers independently screened the titles and abstracts to exclude studies that did not meet the criteria. Then, read through the whole article again to weed out the appropriate research. In addition, the list of references included in the studies was used to search for more relevant studies.

### 2.5 Data extraction

Two researchers (CJL and ZM) compiled data in an Excel spreadsheet to facilitate the data extraction procedure. The primary investigators were also approached to obtain any supplementary relevant information. Information including author(year), study design, participant details, intervention, outcome data, adverse effects, and loss of follow-up were extracted from each study. Any discrepancies in the extracted data were resolved through consensus, with the involvement of a third author (ZZY) when needed.

### 2.6 Risk of bias assessment

The studies’ quality was evaluated employing the Cochrane Risk of Bias assessment tool. The evaluation standards comprised random sequence generation, allocation concealment, blinding of participants and staff, blinding of outcome assessment, incomplete outcome data, selective reporting, and other biases. Two authors (CJL and ZM) independently assessed the risk of bias of included studies and rated each domain as having a low risk of bias, a high risk of bias or an unclear risk of bias.

### 2.7 Statistical analysis

Meta-analysis was performed using Rev. Man 5.4 and R4.3.2 software, with count data expressed as relative risk (*RR*) and 95% *CI*. Continuous variables such as SBP, DBP, TC, TG, LDL-C, HDL-C, SAS, and PSQI scores expressed as mean difference (*MD*) and 95% *CI*. If the Q-test *p* > 0.05 and *I*^2^ < 50%, there was no statistically significant heterogeneity between studies, a fixed-effects model was used for meta-analysis. If *p* < 0.05, *I*^2^ > 50%, there was greater heterogeneity between studies, the random-effects model was used to calculate the composite effect size, and the source of heterogeneity was further analyzed using meta-regression. Publication bias between studies was assessed using funnel plots and Egger’s test, and for those with significant publication bias, the combined effect was analyzed by interpolation using the cut-and-patch method. Statistical significance was set at *p* < 0.05.

## 3 Results

### 3.1 Selection outcomes

**Figure 1:**
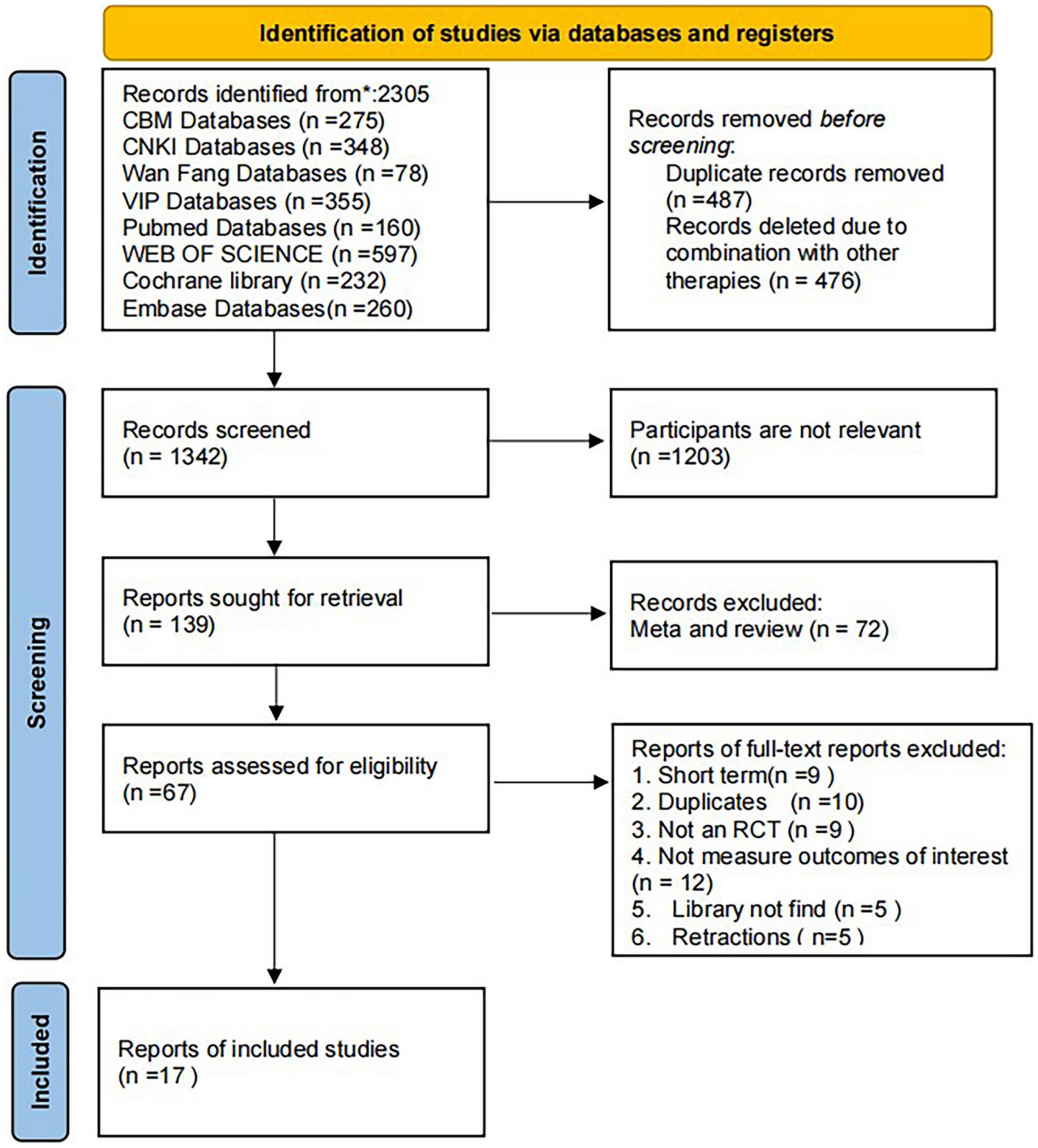
PRISMA flow diagram.

Upon conducting a database search, a total of 2,305 records were retrieved. After applying the inclusion criteria, 17 studies were deemed suitable for inclusion in the systematic review and meta-analysis. The study selection process is presented in Figure 1.

### 3.2 Study characteristics

Seventeen completed trials with 1755 participants who were randomly assigned and met the inclusion criteria. All 17 studies are from China. There was a difference in the health status of the participants between the trials. Two trials recruited healthy participants (25, 26), one trial recruited older adult people with high blood lipids (27), one trial recruited older adult people with pre-diabetes (28), and thirteen trials recruited hypertensive patients.

Follow-up periods varied among the included studies, ranging from 12 weeks (29–32) to 20 weeks (25), 24 weeks (33), 3 months (26, 34), 6 months (35–38), 1 year (28), and 18 months (27), to as long as 30 years in three studies (39–41).

The duration and frequency of Ba Duan Jin practice varied across the 17 studies analyzed. Seven studies (29, 31, 32, 36, 39–41) reported practicing Ba Duan Jin for 13–20 min per session, once or twice daily, five times a week. Among the five studies (30, 34, 35, 37, 38), the practice duration was 30–40 min, once or twice daily, five times a week. In the remaining four studies (25, 26, 28, 33), Ba Duan Jin was performed twice daily for 50–60 min, 5–7 times a week. Among the 17 studies, 10 used the Ba Duan Jin intervention, whereas the remaining 7 studies utilized the Ba Duan Jin-Qigong therapy intervention. Details of the included studies are provided in Supplementary Figure S1.

### 3.3 Assessment of the risks of study bias

**Figure 2:**
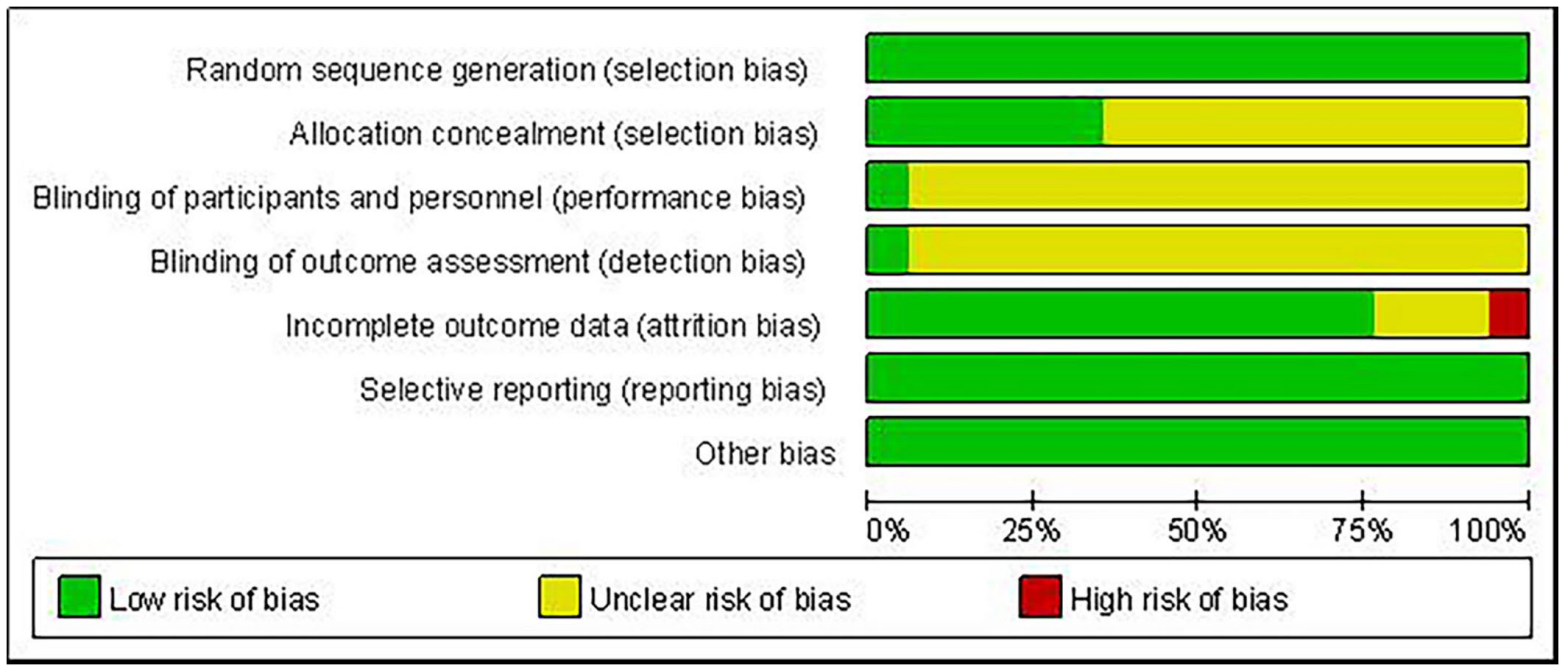
Over risk of bias: review authors’ judgements about each risk of bias item for each included study.

**Figure 3:**
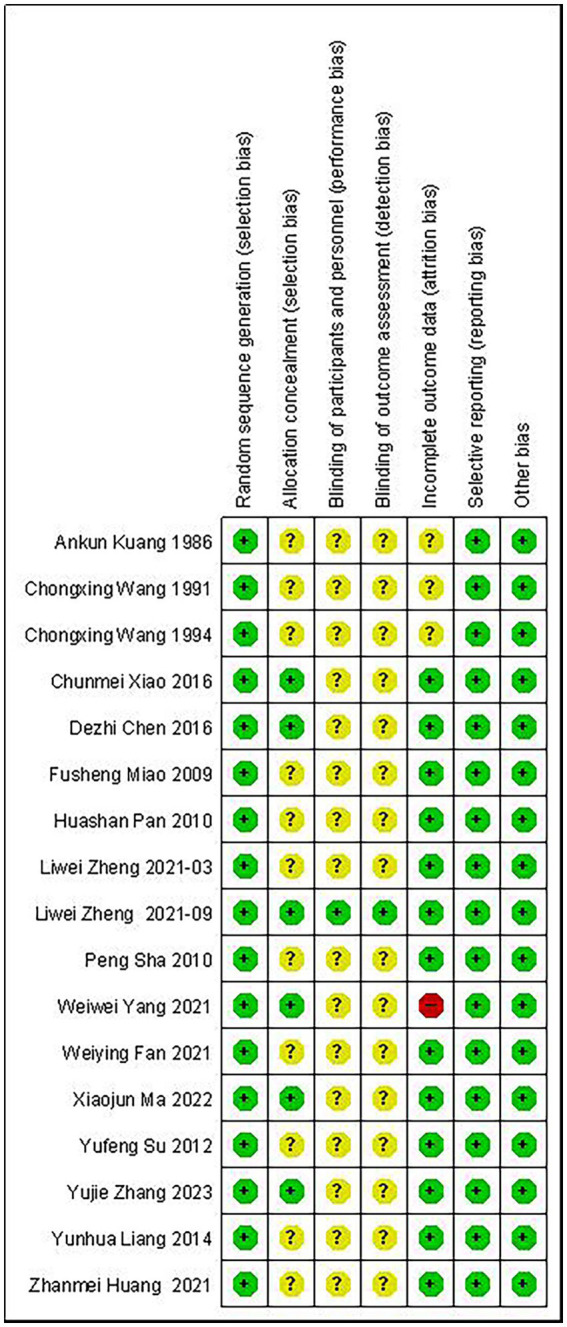
Risk of bias graph: review authors’ judgements about each risk of bias item presented as percentages across all included studies. “+”, low risk; “–”, high risk; “?”, unclear.

Of the 17 RCTs included, 1 had a high risk of bias, 15 had an unclear risk of bias, and 1 had a low risk of bias. The study with high risk of bias was due to missing results because participants failed to complete the entire study during the experiment. Articles with unclear risk of bias were mainly due to insufficient information provided on the study methodology, especially in the areas of random sequence generation, allocation concealment, and participant blinding. In one trial, the research assistant who collected and entered the trial data remained blinded to group allocation throughout the trial, and this was considered to be at low risk of bias. The risk of bias in included studies was demonstrated in Figure 2. The risk of bias in individual trials was shown in Figure 3.

### 3.4 Meta-analysis results

#### 3.4.1 Mortality

Based on the 17 completed included studies, three of them provided statistics on all-cause mortality, stroke mortality, and myocardial infarction (MI) mortality after 20 to 30 years of follow-up (39–41). However, it is unclear whether the Ba Duan Jin-Qigong intervention will continue over a period of 20 to 30 years.

**Figure 4:**
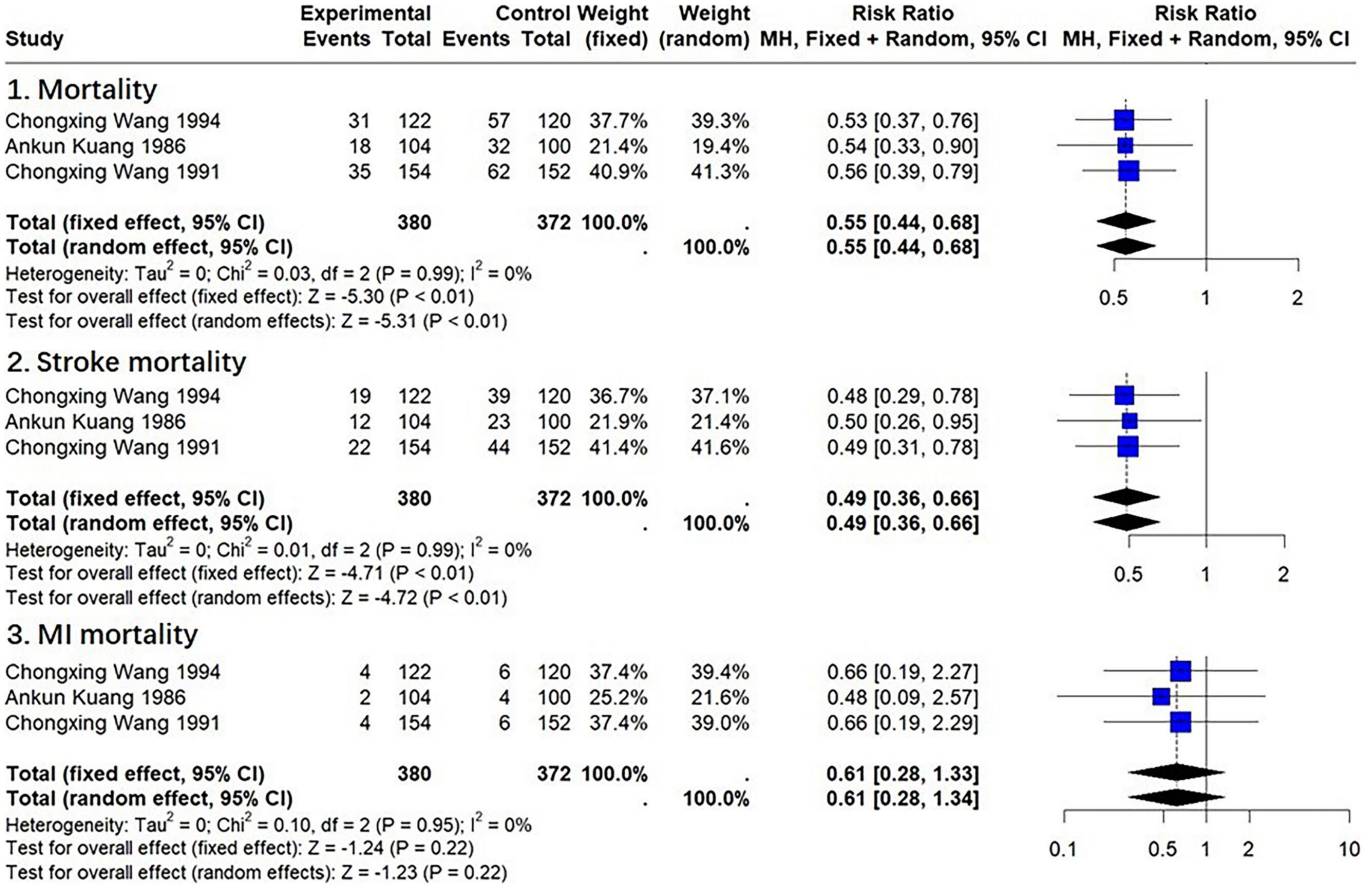
Effect of Ba Duan Jin exercise on mortality.

In three trials (*n* = 752), Ba Duan Jin exercise intervention significantly reduced all-cause mortality (*RR* = 0.55, 95% *CI*: 0.44–0.68, *p* < 0.01). In three trials (*n* = 752), Ba Duan Jin exercise intervention was found to significantly reduce stroke mortality (*RR* = 0.49, 95% *CI*: 0.36–0.66, *p* < 0.01). In three trials (*n* = 752), Ba Duan Jin exercise intervention had no significant effect on mortality from MI (*RR* = 0.61, 95% *CI*: 0.28–1.33, *p* = 0.22) (Figure 4). The funnel plot suggested that there was no significant bias (Supplementary Figure S2). The sensitivity analysis suggested a better model robustness (Supplementary Figure S3).

#### 3.4.2 Blood pressure

**Figure 5:**
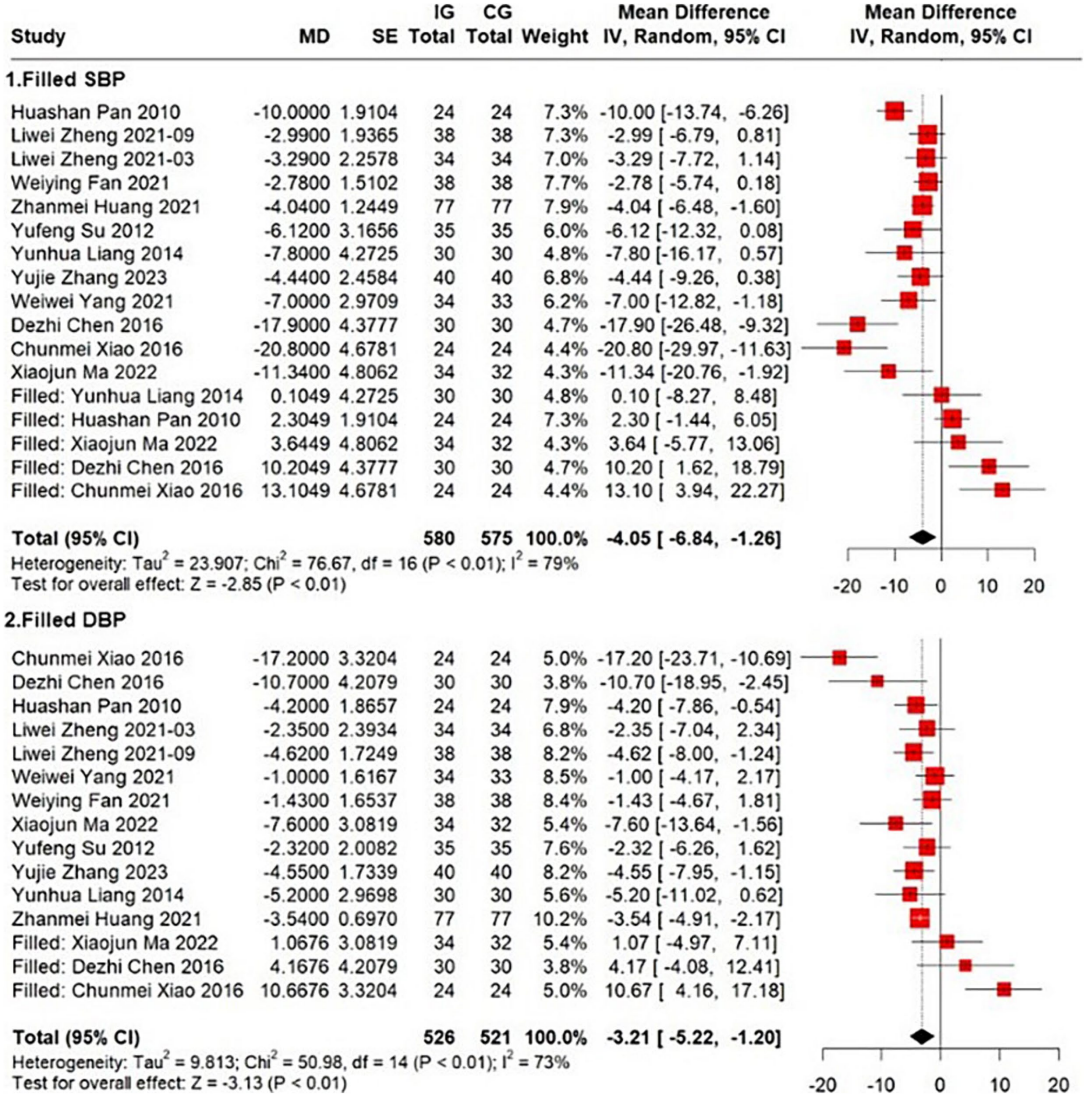
Effect of Ba Duan Jin exercise on SBP & DBP.

Twelve of the 17 included studies measured the effect of the Ba Duan Jin exercise intervention on participants’ blood pressure (26, 28–38). Of the 17 included studies, 12 measured the effect of the Ba Duan Jin exercise intervention on subjects’ blood pressure. Three studies investigated the long-term effects of the Ba Duan Jin exercise intervention on the systolic blood pressure (SBP) of the subjects and therefore only recorded blood pressure values before the start of the experiment and did not carry out blood pressure changes at the end of the intervention (39–41). A pooled analysis revealed a considerable degree of heterogeneity among the studies (*I*^2^ = 67%, *p* = 0.0004), as indicated by Egger’s test (*p* = 0.0129), the funnel plot results suggested a significant publication bias (Supplementary Figure S2). Firstly, the studies were interpolated by the trim and fill method and then combined by a random effects model. The analysis of the results demonstrated a significant benefit of Ba Duan Jin exercise (*MD* = −4.05, 95% *CI* = −6.84 to −1.26, *p* < 0.01) (Figure 5). The funnel plot after interpolation is presented in Supplementary Figure S4. The model demonstrated good robustness (Supplementary Figure S5). Meta-regression with age and number of participants, with female introduced as covariates, revealed that heterogeneity was fully explained (*I*^2^ = 0, *p* = 0.9395, *p* = 0.0008 for age).

A total of twelve trials (*n* = 873) were conducted to assess the effect of Ba Duan Jin exercise on blood pressure. The diastolic blood pressure (DBP) of participants was measured before and after the intervention. However, there was considerable heterogeneity among the studies (*I*^2^ = 60%, *p* = 0.0042). The results of funnel plot suggested possible publication bias (Supplementary Figure S2). The same trim and fill method were employed to interpolate the studies prior to combining them in a random-effects model. The results are presented in Figure 5. The analysis of the results demonstrated a significant benefit of the Ba Duan Jin exercise (*MD* = −3.21, 95% *CI* = −5.22 to −1.20, *p* < 0.01). The funnel plot after interpolation is presented in Supplementary Figure S4. The model demonstrated good robustness (Supplementary Figure S5). A meta-regression incorporating age and the number of female participants as covariates demonstrated that heterogeneity was fully explained (*I*^2^ = 0, *p* = 0.5679). However, age and the number of female participants were not statistically significant in the model. The results indicated that the Ba Duan Jin exercise intervention significantly reduced both SBP and DBP of the participants, thereby demonstrating a positive effect on blood pressure.

#### 3.4.3 Total cholesterol

**Figure 6:**
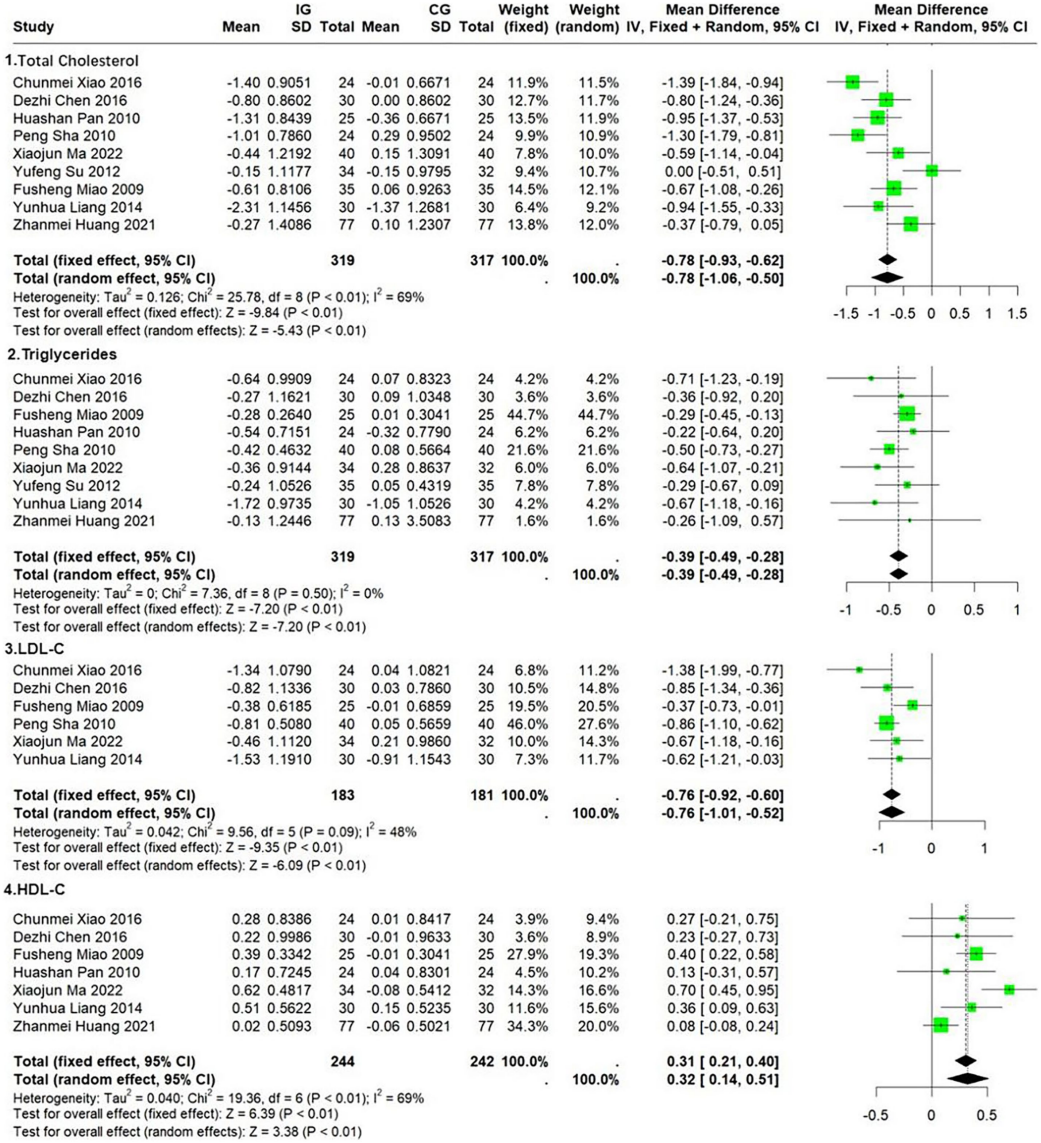
Effect of Ba Duan Jin exercise on TC & TG & LDL-C & HDL-C.

Eight trials (*n* = 588) were conducted to assess the impact of the Ba Duan Jin exercise intervention on participants’ total cholesterol (TC). The results demonstrated significant heterogeneity (*I*^2^ = 69%, *p* = 0.0011). However, publication bias was not statistically significant (Egger’s test *p* = 0.9985, Supplementary Figure S2). The effect sizes of the individual trials are presented in Figure 6. Six trials demonstrated a statistically significant reduction in total cholesterol with the Ba Duan Jin exercise intervention, while the remaining trial did not show a significant reduction in total cholesterol with the Ba Duan Jin exercise intervention (26, 34). Pooled analyses demonstrated that the Ba Duan Jin exercise intervention significantly reduced total cholesterol levels in participants (*MD* = −0.78, 95% *CI* = −1.06 to −0.50, *p* < 0.01). Heterogeneity could be partially explained by introducing age and female for meta-regression (*I*^2^ = 29.93%, *p* = 0.2322). The sensitivity analysis suggested a better model robustness (Supplementary Figure 6).

#### 3.4.4 Triglycerides

The results for triglycerides (TG) were similar to those for total cholesterol. No significant heterogeneity (*I*^2^ = 0, *p* = 0.50) or bias (Egger’s test, *p* = 0.2988, Supplementary Figure 2) were identified. The pooled results of nine studies (*n* = 636) indicated that the Ba Duan Jin exercise intervention had a significant effect on triglyceride levels, with a mean difference of −0.78 (95% *CI* −0.93 to −0.62, *p* < 0.01). The intervention was found to have a significant effect on triglyceride levels, with a mean difference of −0.78 (95% *CI* −0.93 to −0.62, *p* < 0.01) (Figure 6). This could have a beneficial effect on the cardiovascular system. The model demonstrated good robustness (Supplementary Figure S6).

#### 3.4.5 Low density lipoprotein cholesterol

The results for low density lipoprotein cholesterol (LDL-C) were comparable to those for total cholesterol (TC) and triglycerides (TG). There was no evidence of heterogeneity (*I*^2^ = 48%, *p* = 0.09) or bias (Egger’s test, *p* = 0.9001, Supplementary Figure 2). The pooled results from six studies (*n* = 364) indicated that the Ba Duan Jin exercise intervention significantly reduced participants’ LDL cholesterol levels (Figure 6), which may have beneficial effects on the cardiovascular system (MD = −0.76, 95% CI = −0.92 to −0.60, *p* < 0.01). The model demonstrated good robustness (Supplementary Figure S6).

#### 3.4.6 High density lipoprotein cholesterol

Basic research has shown that high density lipoprotein cholesterol (HDL-C) functions as an anti-atherosclerotic and CVD preventive agent due to its ability to transport cholesterol in reverse, as well as its anti-inflammatory and antioxidant properties. There was considerable heterogeneity (*I*^2^ = 69%, *p* = 0.0036) but no significant bias (Egger’s test, *p* = 0.7870, Supplementary Figure S2) among the seven studies (*n* = 486). The combined results illustrated that the octopus exercise intervention significantly increased the participants’ HDL-C levels and had a positive effect on the cardiovascular system (*MD* = 0.32, 95% *CI* = 0.14–0.51, *p* < 0.01) (Figure 6). A meta-regression analysis was conducted with age and the number of female participants as covariates. This analysis demonstrated that heterogeneity was partially explained (*I*^2^ = 29.93%, *p* = 0.2322). The model demonstrated good robustness (Supplementary Figure S6).

#### 3.4.7 Quality of life

**Figure 7:**
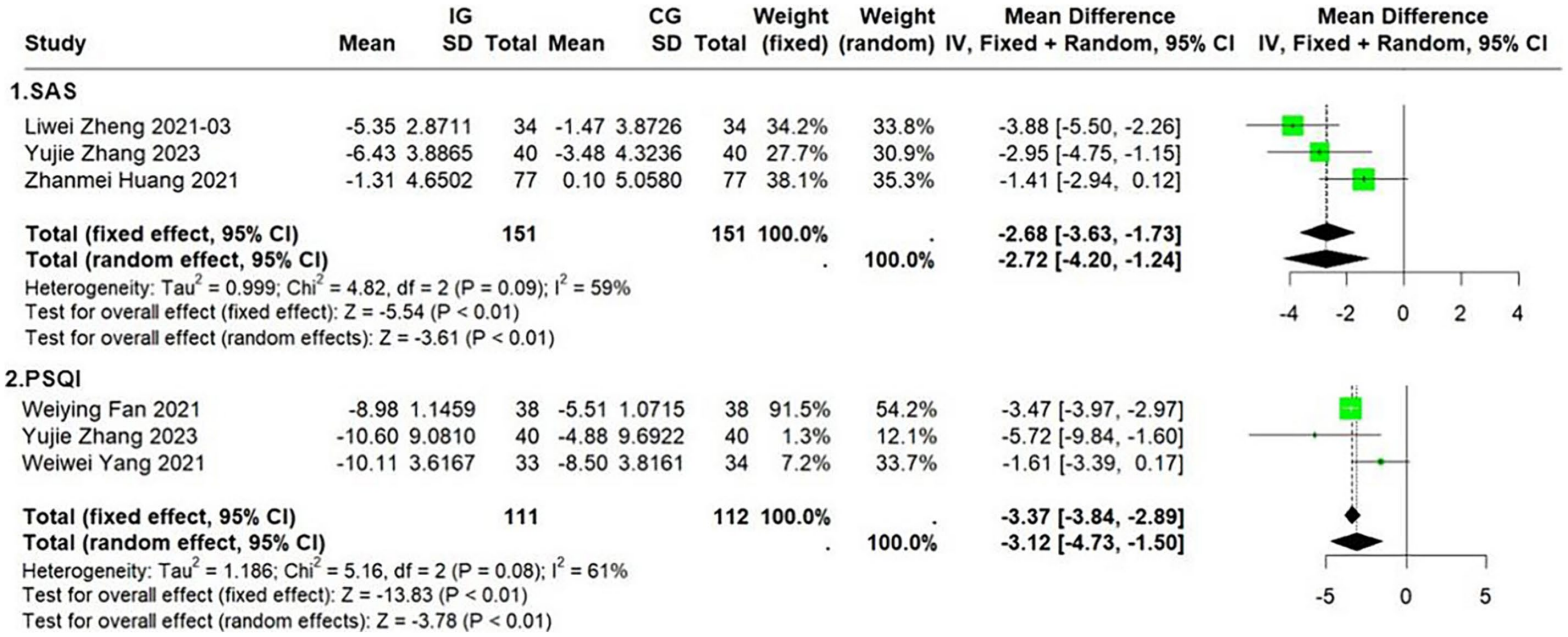
Effect of Ba Duan Jin exercise on SAS & PSQI.

Five of the 17 trials assessed the effects of the Ba Duan Jin exercise intervention on participants’ anxiety and sleep quality. Three trials (*n* = 223) used the Self-Assessment Scale for Anxiety (SAS) to assess participants before and after the intervention. There was no significant heterogeneity between studies (*I*^2^ = 61%, *p* = 0.0757) and no significant bias (Supplementary Figure S2). The results demonstrated that the Ba Duan Jin exercise intervention significantly reduced participants’ anxiety (*MD* = −3.37, 95% *CI* = −3.84 to −2.89, *p* < 0.01) (Figure 7). The results were robust (Supplementary Figure S7).

Three trials (*n* = 302) assessed participants’ sleep quality before and after the intervention using the Pittsburgh Sleep Quality Index scale (PSQI). There was no significant inter-study heterogeneity (*I*^2^ = 59%, *p* = 0.0896) or bias (Supplementary Figure S2). The results demonstrated that the Ba Duan Jin exercise intervention significantly improved participants’ sleep quality (*MD* = −2.68, 95% *CI* = −3.63 to −1.73, *p* < 0.01) (Figure 7). The results were robust (Supplementary Figure S7).

## 4 Discussion

Although there is considerable heterogeneity in the results of most studies, the present study’s systematic review and meta-analysis of clinical research trials suggests that the Ba Duan Jin exercise intervention reduces mortality, improves cardiovascular health, and enhances quality of life in patients with cardiovascular systems. Furthermore, the study indicates that the Ba Duan Jin exercise intervention can have a positive effect on cardiovascular disease, regardless of the frequency or duration of the exercise.

Numerous research has indicated a strong association between hypertension and a higher risk of CVD, independent of other risk factors (42). High blood pressure is the primary modifiable risk factor globally, contributing to increased all-cause morbidity and mortality (43). Lifestyle modifications such as increased physical activity have proven to effectively lower blood pressure and prevent the onset of hypertension and its cardiovascular disease implications (44). Ba Duan Jin, a form of physical activity, has been shown to have significant antihypertensive benefits for individuals with hypertension, and it is well-liked by patients because it has minimal side effects (22, 45, 46). Additionally, a study has indicated that this intervention can effectively modulate cerebral hemodynamics, reduce blood pressure, and enhance the overall risk of ischemic stroke in older adults (47). In 12 of the 17 studies, participants were diagnosed with essential hypertension. Based on the results of our meta-analysis, it is evident that Ba Duan Jin exercise therapy has a significant antihypertensive effect on hypertension. Additionally, it may also have a positive impact on various cardiovascular and cerebrovascular susceptibility factors. These findings suggest that Ba Duan Jin exercise therapy could potentially play a role in preventing stroke and improving the prognosis of hypertension.

Blood lipids are significant predictors of the risk of atherosclerosis, coronary heart disease, and ischemic stroke (48–50). Dyslipidemia, which is characterized by abnormal lipid profiles, can lead to various health issues such as metabolic syndrome and cardiovascular disease (51–54). These profiles comprise high levels of total cholesterol (TC), high levels of triglycerides (TG), low levels of high-density lipoprotein cholesterol (HDL-C), and elevated levels of low-density lipoprotein cholesterol (LDL-C) (55). This study indicates that Ba Duan Jin exercise intervention can effectively reduce blood lipids, improve lipid metabolism, and alleviate clinical symptoms in patients with hyperlipidemia. Therefore, Ba Duan Jin exercise is highly recommended for preventing and treating hyperlipidemia. Furthermore, a study has demonstrated that the Ba Duan Jin exercise can significantly decrease the plasma levels of TC and LDL-C, while increasing the plasma level of HDL-C in healthy individuals. Additionally, the Ba Duan Jin exercise aids in regulating lipid metabolism in patients. These findings are in line with the results of the current study (56).

Sleep disorders have been associated with the development and advancement of various illnesses, including cardiovascular disease, depression, and cancer (57–59). Several factors can have a detrimental effect on sleep quality, such as stress and anxiety (60). Increasing evidence suggests that physical activity can significantly improve sleep quality (61). For instance, a meta-analysis has confirmed that qigong and tai chi exercises can reduce cardiometabolic risk factors, such as psychosocial stress, poor sleep quality, and weight gain. These exercises are particularly suitable for older individuals (62). In this study, we found that Ba Duan Jin exercise therapy can effectively reduce blood pressure and improve sleep quality. It has significant efficacy in treating hypertension with sleep disorders.

To our knowledge, no other systematic review including only randomized controlled trials has been conducted with the specific aim of examining the effects of Ba Duan Jin in adults for the primary prevention of cardiovascular disease (CVD). Therefore, it is difficult to make comparisons with other similar studies at this time. The focus of this review was specifically on the Ba Duan Jin exercise intervention in order to minimize the influence of other behavioral interventions on the results. This approach was taken to narrow down the number of trials eligible for inclusion. However, it is important to note that the limited number of trials, along with their methodological limitations and unclear risk of bias, significantly restrict the conclusions that can be drawn from this review.

## 5 Strengths and limitations

This is the first meta-analysis examining the effects of Ba Duan Jin exercise intervention for the primary prevention of cardiovascular diseases. Our evidence may provide researchers with new ideas and promote more experimental protocols. This study can encourage the public to choose effective and appropriate exercise methods to prevent cardiovascular diseases.

The current review indicates that further trials are required to elucidate the potential benefits of Ba Duan Jin exercise therapy for cardiovascular disease (CVD) risk factors. Furthermore, as all the literature included was conducted in China, there is a lack of data support from validated clinical trials in countries other than China, which may have limited the generalization of the results.

## 6 Conclusion

This conclusion is divided into two main sections.

The first section focuses on the implications for practice. At present, the evidence base for Ba Duan Jin exercise therapy in the context of primary CVD prevention is limited. The trials included in this review were few in number for relevant outcomes and small in sample size, and were at significant risk of bias. Consequently, there is very low confidence in the validity of the results. The clinical event data provided by three trials many years after completion of the trials as originally designed may not be attributable to the intervention, and thus no firm conclusions can be drawn on the basis of their findings. Similarly, alterations in cardiovascular disease (CVD) risk factors may be attributable to suboptimal trial conduct. Consequently, no definitive conclusions can be drawn.

The results of our study demonstrated that Ba Duan Jin exercise has a beneficial effect on patients with essential hypertension through a meta-analysis and systematic evaluation of existing clinical trials. Furthermore, it has been demonstrated to enhance lipid profiles and cardiovascular function in older adult patients with hyperlipidemia. These findings indicate that Ba Duan Jin exercise therapy may be a promising intervention for the primary prevention of cardiovascular disease. In order to ascertain whether Ba Duan Jin exercise represents an efficacious lifestyle intervention for the prevention of CVD, it is necessary to await the publication of further trial evidence derived from methodologically rigorous, adequately powered, long-term trials. It is therefore premature to make any recommendations regarding practice at this stage.

The second section focuses on the implications for research. The current research landscape shows a limited number of RCTs focused solely on evaluating the effectiveness of Ba Duan Jin exercise for the primary prevention of CVD. There is a particular scarcity of large, rigorously conducted RCTs with extended follow-up periods that specifically investigate the effectiveness of Ba Duan Jin exercise for the prevention of major CVD events and CVD risk factors. Additionally, there is a lack of evidence regarding the effectiveness of Ba Duan Jin exercise in populations outside of China. Furthermore, there is a notable absence of information on the incidence of Type 2 Diabetes (T2D) in relation to the intervention, as well as any potential adverse effects and costs associated with the implementation of Ba Duan Jin exercise.

## Data availability statement

The original contributions presented in the study are included in the article/Supplementary material, further inquiries can be directed to the corresponding author.

## Author contributions

JC: Methodology, Writing – original draft, Writing – review & editing, Formal analysis, Investigation. MZ: Methodology, Writing – original draft, Formal analysis, Investigation, Writing – review & editing. YW: Data curation, Formal analysis, Investigation, Writing – original draft. ZZ: Conceptualization, Data curation, Software, Writing – original draft. SG: Data curation, Formal analysis, Writing – original draft. YZ: Funding acquisition, Writing – review & editing.
