# Supplementary material for: The effect of Ba Duan Jin exercise intervention on cardiovascular disease: a meta-analysis of randomized controlled trials
Source: Front Public Health. 2024 Aug 6;12:1425843. doi: 10.3389/fpubh.2024.1425843 (PMC11333314; doi:10.3389/fpubh.2024.1425843)
Supplement: Supplementary file 1 [file Data_Sheet_1.PDF]

Supplementary Material

1 Supplementary Figures and Tables

1.1 Supplementary Figures

**Author (year)**	Huashan Pan 2010
**Method**	RCT<br>All 48 cases were Guangzhou permanent residents . A total of 48 grade 1 hypertension old patients were randomly divided into 2 groups (n=24) :control group, exercise group.
**Participants**	The control group was treated with conventional medication (western medicine was mainly thiazide diuretics, and Chinese medicine was mainly Tianma Hook Teng Drink), while the experimental group was treated with conventional medication and assisted with Ba duan Jin exercise for 24 weeks. The frequency of exercise is 5 days a week, 1 exercise in the morning and 1 exercise in the evening, 2 repetitions of each exercise, with a 2-minute rest between each repetition, together with the preparatory activity at the beginning and the finishing movement at the end, an exercise of about 45 minutes.
**Interventions**	24 weeks
**Outcomes**	Blood pressure, TC, TG,HDL-C, serum insulin and blood glucose
**Author (year)**	Liwei Zheng 2021-09
**Method**	RCT<br>Elderly patients with hypertension with debility who attended the outpatient clinic of the Department of Cardiovascular Medicine at the Affiliated People's Hospital of Fujian University of Traditional Chinese Medicine from June to December 2020 were selected for the study.<br>A total of 67 elderly hypertensive outpatients with frailty were randomized into an intervention group(n=33) and a control group(n=34).
**Participants**	The control group used conventional treatment and health education, including medication care, diet management, blood pressure monitoring and psychological counselling. In the intervention group, a 12-week intervention of Ba Duan Jin was carried out on this basis.5 d of continuous practice per week, 2 sessions per day of 13 min each, with a 5 min break between sessions, for a total of 12 weeks of intervention.
**Interventions**	12 weeks
**Outcomes**	The frailty score, SF-12 score, the gait speed, grip strength and blood pressure
**Author (year)**	Fusheng Miao 2009
**Method**	RCT<br>Fifty middle-aged and elderly patients with hyperlipidaemia were the subjects of this study, none of them had clinically diagnosed endocrine diseases or family history of genetic disorders, and they did not participate in regular physical exercise and did not take lipid-lowering drugs.
**Participants**	Baduan jin group: Each exercise was 50-60 min, 5-7 times/week for 18 months.<br>The control group remained unchanged according to their normal lifestyle.
**Interventions**	18 months
**Outcomes**	TG,TC, HDL-C, LDL-C, ApoA1, ApoB, ApoA1/B
**Author (year)**	Liwei Zheng 2021-03
**Method**	RCT
	A total of 64 elderly patients with EH with insomnia attending the outpatient clinic of the Department of Cardiovascular Medicine at the Affiliated People's Hospital of Fujian University of Traditional Chinese Medicine from March 2019 to October 2019 were used as study subjects.
**Participants**	Sixty-eight cases of elderly patients with primary hypertension and insomnia were randomly divided into two groups, the control group was treated conventionally, and the intervention group was intervened with Ba Duan Jin exercise intervention on this basis for a total of 12 weeks.<br>Exercise 5 days a week, once a day, practicing 2 times each time, about 13 minutes per practice, with a 5-minute rest between the 2 practices. The intervention lasts for a total of 12 weeks.
**Interventions**	12 weeks
**Outcomes**	Pittsburgh Sleep Quality Index (PSQI) score, salivary cortisol levels, blood pressure values, heart rate values

Supplementary Figure 1(a). Basic characteristics of the included literature.

**Author (year)**	Weiying Fan 2021
**Method**	RCT
	A total of 76 elderly patients with essential hypertension accompanied by anxiety and depression who visited the cardiovascular department outpatient clinic of the Affiliated People's Hospital of Fujian University of Traditional Chinese Medicine from October 2019 to July 2020 were randomly divided into an intervention group and a control group, with 38 cases in each group.
**Participants**	Conventional treatment and health education are adopted.<br>Intervention Group：implementing the Ba Duan Jin exercise intervention on the basis of the control group.<br>Exercise plan: Exercise 5 days a week, practice 2 times a day, 13 minutes each time, with a 5-minute break in between, for a total of 12 weeks.
**Interventions**	12 weeks
**Outcomes**	Self-Rating Anxiety Scale (SAS), Self-Rating Depression Scale (SDS), abbreviated version of the Quality of Life Scale SF-12 scores, and blood pressure levels.
**Author (year)**	Zhanmei Huang 2021
**Method**	RCT<br>Select the Health Management Physical Examination Center of the First Affiliated Hospital of Nanchang UniversityThose who meet the following inclusion criteria between January 2020 and January 2021A total of 154 patients with grade 1 hypertension were randomly divided into an observation group and a control group with 77 patients each after signing the informed consent form.
**Participants**	Patients in both groups were routinely guided on a low-salt and low-fat diet and observed<br>The observation group received Baduanjin exercise twice a day (7:00 a.m. and 18:00 p.m.) for 30 min each time for 5 d per week for 3 months. The control group received no other intervention.
**Interventions**	3 months
**Outcomes**	HbAlc、TC、TG、LDL-C、HDL-C,Hamilton Anxiety Rating Scale, Pittsburgh Sleep Quality Index
**Author (year)**	Yufeng Su 2012
**Method**	RCT<br>Select 70 elderly women aged 55-69 from Xianlin and Gulou communities in Nanjing, Jiangsu Province, with an average age of 61.4±5.6 years.
**Participants**	The subjects were randomly divided into two groups: the training group and the control group, with 35 subjects in each group. There were no significant differences in age between the two groups of subjects.<br>The practice group receives a week of Easy Muscle Tendon Change Classic training before the experiment, and begins the experiment officially after mastering the movements.Training time and frequency: Practice the Yi Jin Jing five times a week, with each session lasting 60 minutes, for a continuous period of 3 months.<br>The control group did not participate in any planned collective or individual exercise throughout the entire 3-month period.
**Interventions**	3 months
**Outcomes**	Blood pressure, lung capacity, sit-and-reach, grip strength, back strength, reaction time, single-leg stance with eyes closed, TC, TG, HDL, LDL
**Author (year)**	Yunhua Liang 2014
**Method**	RCT<br>Sixty patients with primary hypertension who were hospitalized in The Second Clinical Medical College of Jinan University from July 2011 to December 2012 were selected. They were classified according to the 1999 Chinese guidelines for the treatment of hypertension, all of whom met the diagnostic criteria for stage 1 or stage 2 hypertension, and were excluded from secondary hypertension, diabetes, pulmonary heart disease, cerebrovascular disease, nephrotic syndrome, and other diseases that affect blood lipids, as well as patients with contraindications to exercise. They were randomly divided into a control group and an experimental group, with 30 cases in each group.
**Participants**	In the control group, there were 18 males and 12 females, with an average age of (55.7±8.8) years and a duration of illness of (4.7±3.2) years. In the experimental group, there were 20 males and 10 females, with an average age of (54.8 ±7.6) years and a duration of illness of (4.3±3.0) years. Both groups received routine nursing care and conventional drug treatment. Patients in the control group walked twice a day, 20 minutes each time, while the experimental group received intervention treatment with the Eight Section Brocade Qigong exercise.Practice 5 days a week, 2 times a day, once in the morning and once in the afternoon, for 20 minutes each time, and continue exercising for 6 months, during which other sports activities are suspended.
**Interventions**	6 months
**Outcomes**	TG、TC、LDL-C and HDL-C

Supplementary Figure 1(b). Basic characteristics of the included literature.

**Author (year)**	Peng Sha 2010
**Method**	RCT<br>After physical examination screening, 80 residents of Mingde Community were selected as the experimental subjects, and were randomly divided into an exercise group and a control group, with 40 people in each group, all of whom had no regular exercise experience. The subjects were all from the same city, with similar living conditions. During the experiment, the subjects' diet and other lifestyle habits were not different from before the experiment. All subjects had no history of bone metabolism diseases, cerebrovascular diseases, or family medical history.
**Participants**	Control group no exercise intervention.<br>Exercise group performs Wuqinxi Qigong Exercise intervention. The entire experiment process lasts for 20 weeks, divided into two stages. The first 2 weeks are for learning, and the following 18 weeks are for consolidation and practice. Participants will collectively follow the accompaniment music for exercise from 7:00 to 8:00 every morning under the guidance of the counselor (specially trained), for 60 minutes, 5 days a week.
**Interventions**	18 weeks
**Outcomes**	Blood lipid indicators: TC, TG, HDL-C, LDL-C, APOA1, APOB. Open-eye static balance, closed-eye static balance
**Author (year)**	Chongxing Wang 1994
**Method**	RCT<br>From 1958 to 1963, 242 patients with essential hypertension were treated with qigong in the outpatient clinic and ward of Shanghai Institute of Hypertension according to the design plan, all of whom were male, with an average age of 48.21±5.60 years old at the time of treatment.
**Participants**	242 hypertensive patients were randomly divided into two groups: qigong combined with regular medication group (referred to as qigong group, n=122) and simple regular medication group (referred to as control group, n=120).<br>During the follow-up period, the patients in the two groups were regularly taken antihypertensive drugs according to a unified design plan, and the initial variety and dose were completely consistent. From 1958 to 1969, he took reserpine, hydralazine, and dihydrogram urinary plugs, half a tablet each time, three times a day; From 1970 to 1980, he took one tablet each time, three times a day; From 1981 to 1992, he took one tablet at a time, three times a day. During the follow-up period, the dose was increased or decreased according to the unified floating medication regimen.<br>Qigong exercise is mainly based on sitting meditation, combined with standing postures, supplemented by guidance and massage, 1-2 times a day, 20-30 minutes each time.<br>The control group did not perform Qigong exercises
**Interventions**	30 years
**Outcomes**	The overall mortality rate, the stroke mortality rate and the MI mortality rate
**Author (year)**	Ankun Kuang 1986
**Method**	RCT<br>204 patients were randomly assigned to receive either qigong combined with low-dose antihypertensive drugs (qigong group, n=104) or low-dose antihypertensive drugs alone (control group, n=100) during the treatment.
**Participants**	Two groups take the same variety of low-dose antihypertensive drugs with a unified floating dosage standard for dose adjustment.<br>Qigong group:Qigong exercise is mainly based on sitting meditation, combined with standing postures, supplemented by guidance and massage, 1-2 times a day, 20-30 minutes each time.<br>The control group did not perform Qigong exercises
**Interventions**	20 years
**Outcomes**	The overall mortality rate, the stroke mortality rate and the MI mortality rate
**Author (year)**	Chongxing Wang 1991
**Method**	RCT<br>306 patients were randomly assigned to receive either qigong combined with low-dose antihypertensive drugs (qigong group, n=154) or low-dose antihypertensive drugs alone (control group, n=152) during the treatment.
**Participants**	Two groups take the same variety of low-dose antihypertensive drugs with a unified floating dosage standard for dose adjustment.<br>Qigong group:Qigong exercise is mainly based on sitting meditation, combined with standing postures, supplemented by guidance and massage, 1-2 times a day, 20-30 minutes each time.<br>The control group did not perform Qigong exercises
**Interventions**	25-30 years
**Outcomes**	Total cumulative deaths, deaths due to complications and comorbidities of hypertension, incidence of hypertensive stroke, and deaths and the MI mortality rate

Supplementary Figure 1(c). Basic characteristics of the included literature.

**Author (year)**	Yujie Zhang 2023
**Method**	RCT<br>From June 2021 to June 2022, 80 elderly patients with hypertension in the Department of Hypertension of the Affiliated Hospital of Traditional Chinese Medicine of Xinjiang Medical University were selected as the study subjects and randomly divided into control group (n=40) and observation group (n=40).
**Participants**	The control group received conventional intervention, while the observation group received Baduanjin exercises on the basis of the control group. Frequency of training: The patients performed Baduanjin exercise 5d per week, 1 time/day.The training time was about 30-40 min per session, and both groups of elderly hypertensive patients were intervened continuously for 6 months.
**Interventions**	6 months
**Outcomes**	SBP, DBP, SAS and SDS
**Author (year)**	Weiwei Yang 2021
**Method**	RCT<br>Conveniently selected 70 hypertensive patients who were treated in a community hospital outpatient department as the research subjects, and the diagnostic criteria for hypertension need to comply with the regulations in the "Chinese Hypertension Treatment Guidelines 2018". The researchers used a random number table to randomly divide the included subjects into an observation group and a control group (35 cases each).<br>Inclusion criteria: (1) Grade 1 or Grade 2 hypertension; (2) Age ≥ 60 years; (3) Taking Lacidipine and (or) Losartan potassium as antihypertensive drugs; (4) Self-rating Anxiety Scale (SAS) and Self-rating Depression Scale (SDS) scores both > 50, and have not taken any anti-anxiety or antidepressant drugs; (5) Education level of primary school or above, with normal reading and expression ability; (6) Able to engage in normal physical activity; (7) Patients must have a good understanding of the trial and sign an informed consent form before enrollment.<br>Exclusion criteria: (1) Patients with severe heart, kidney, liver, or other diseases; (2) Secondary hypertension; (3) Patients with severe mental disorders or personality disorders.
**Participants**	The control group received routine care, patiently guided patients to take antihypertensive medications correctly, and actively provided health education on hypertension knowledge to family members and patients. The observation group, based on the intervention of the control group, conducted a 12-week Baduanjin exercise, exercising for 30 minutes each time, twice a day, 5 times a week.
**Interventions**	12 weeks
**Outcomes**	SBP, DBP, SDS、SAS,GSES
**Notes:**	During the course of the study, 2 participants in the observation group voluntarily withdrew from the study due to personal reasons; and 1 participant in the control group dropped out of the study due to loss to follow-up in the 4th week. Therefore, a total of 33 participants were included in the observation group and 34 participants in the control group for statistical analysis.
**Author (year)**	Dezhi Chen 2016
**Method**	RCT<br>Sixty individuals with essential hypertension (mean age: 66.3 ± 5.8 years) were randomized into two groups: 30 to a Mawangdui Daoyinshu Qigong (MDQ) group and 30 to a control group.
**Participants**	MDQ was performed five times per week for 6 months; this included a warm-up (5 minutes), the Qigong exercise (34 minutes), and a cool-down (5 minutes). Controls received no intervention.
**Interventions**	6 months
**Outcomes**	Weight (kg), body mass index (BMI), systolic blood pressure (SBP), diastolic blood pressure (DBP), stroke volume (SV), cardiac output (CO), cardiac index (CI), total peripheral resistance (TPR), high-density lipoprotein cholesterol (HDL-C), low-density lipoprotein cholesterol (LDL-C), total cholesterol (TC), triglycerides (TG), fasting glucose (FG), serum nitric oxide (NO) and plasma endothelin-1 (ET-1)
**Author (year)**	Chunmei Xiao 2016
**Method**	RCT<br>Forty-eight individuals with essential hypertension (mean age 65.6 ± 7.8) were randomized to two groups: 24 to a Ba duan jin Qigong Group (BQG) and 24 to a control group (CG).
**Participants**	The BQG trained in Ba duan jin (Figure 1) five times per week for 6 months. Sessions included a 5-minute warm-up, 30 minutes of Qigong exercise, and a 5-minute cool-down. The CG received no intervention.
**Interventions**	6 months
**Outcomes**	Weight, waist circumference, systolic BP (SBP), diastolic BP (DBP), high-density lipoprotein cholesterol (HDL-C), low-density lipoprotein cholesterol (LDL-C), total cholesterol (TC), triglycerides, fasting glucose, serum nitric oxide (NO) and plasma endothelin-1 (ET-1)
**Author (year)**	Xiaojun Ma 2022
**Method**	RCT<br>A total of 66 subjects completed this study. Their average age was 59.35 ± 4.56 years. There were 34 participants in the BDJ group, 32 participants in the control group.
**Participants**	Among the participants in the study, 73(74.5%) were overweight or obese (BMI ≥ 24 kg/m2), 91(92.9%) exhibited abdominal obesity (WHtR > 0.5), 32(32.7%) were using antihypertensive drugs, and 31(31.6%) were currently smoking. Before the exercise intervention, some participants changed their dosage of antihypertensive drugs or smoking status, but during the intervention period, the numbers of participants who took antihypertensive drugs or smoked did not change; no participants took drugs that affected blood glucose or blood lipid profiles.
**Interventions**	12 months
**Outcomes**	Blood glucose, blood lipid profiles, body shape, blood pressure and 10-year risk of ASCVD

Supplementary Figure 1(d). Basic characteristics of the included literature.

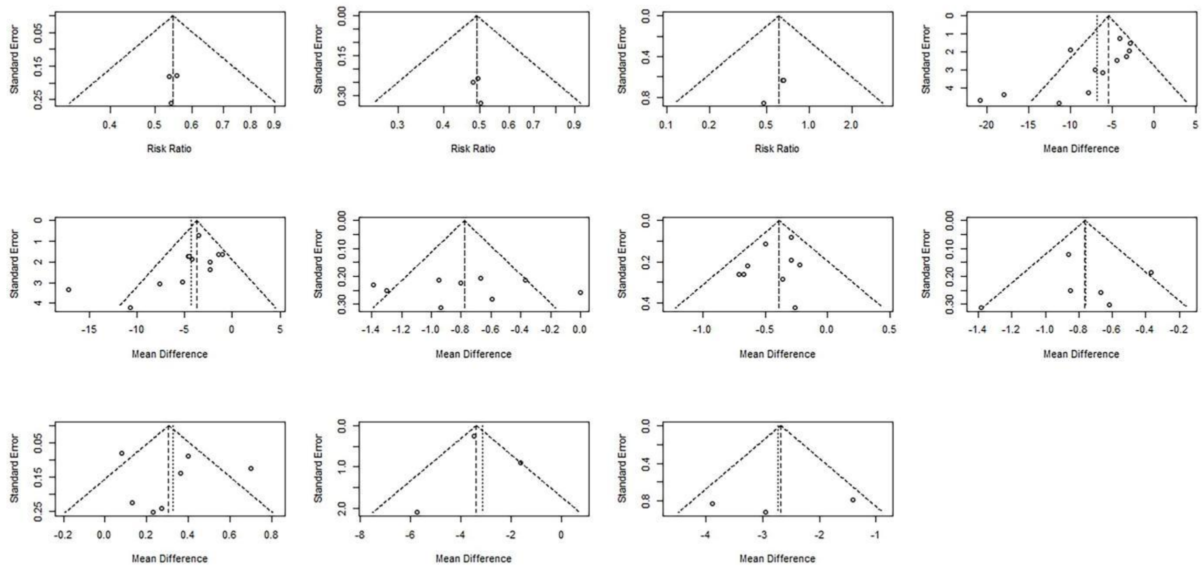

Supplementary Figure 2. Funnel Chart-Sensitivity analysis.

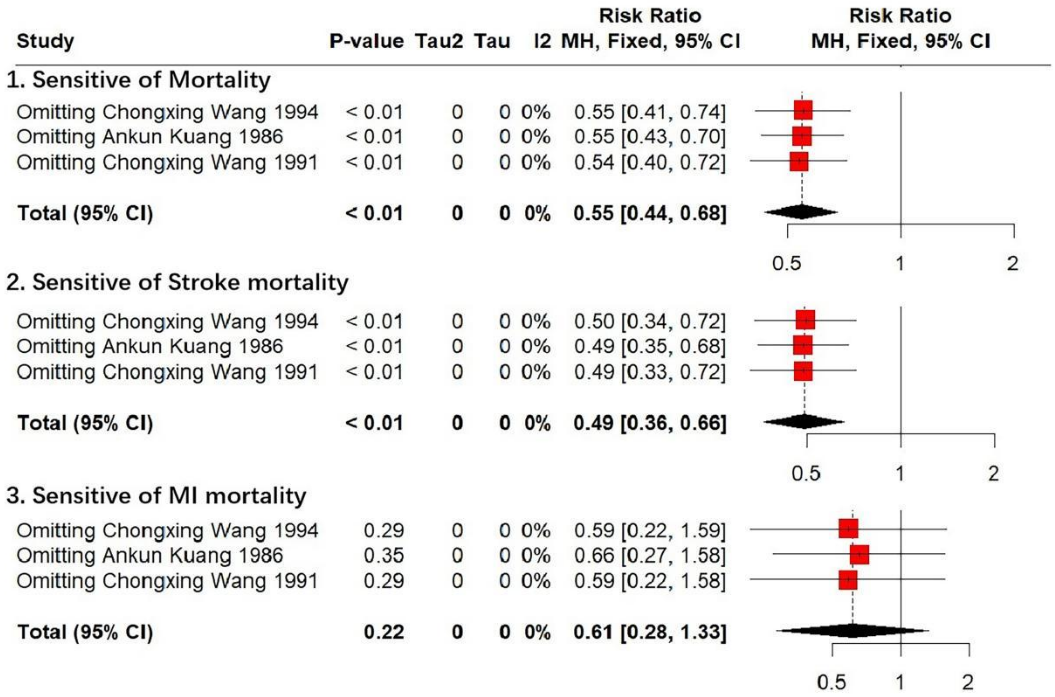

Supplementary Figure 3. Sensitive of Mortality.

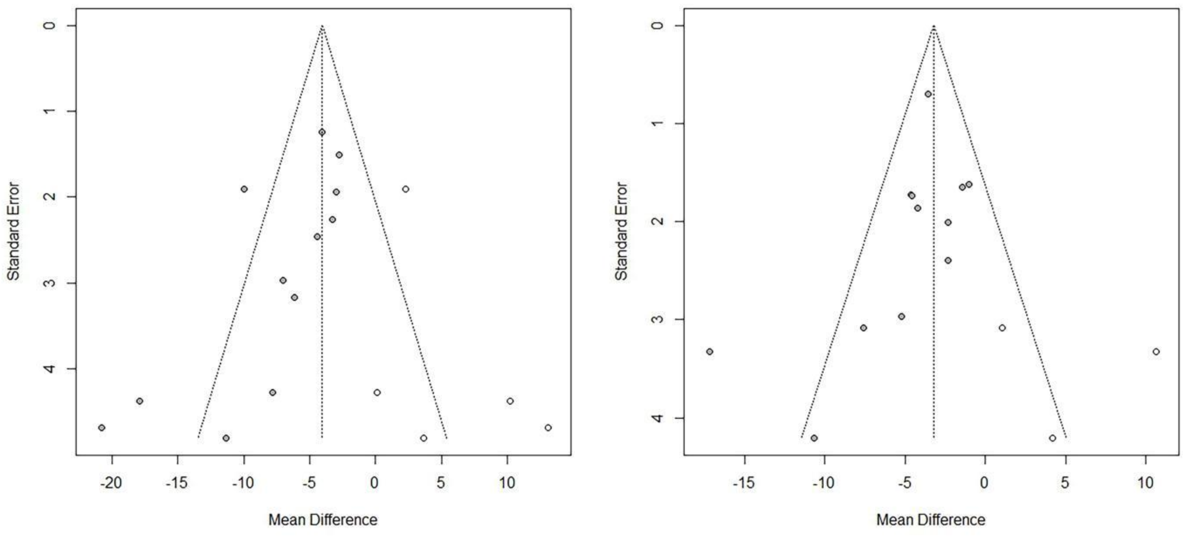

Supplementary Figure 4. Funnel of filled SBP&DBP.

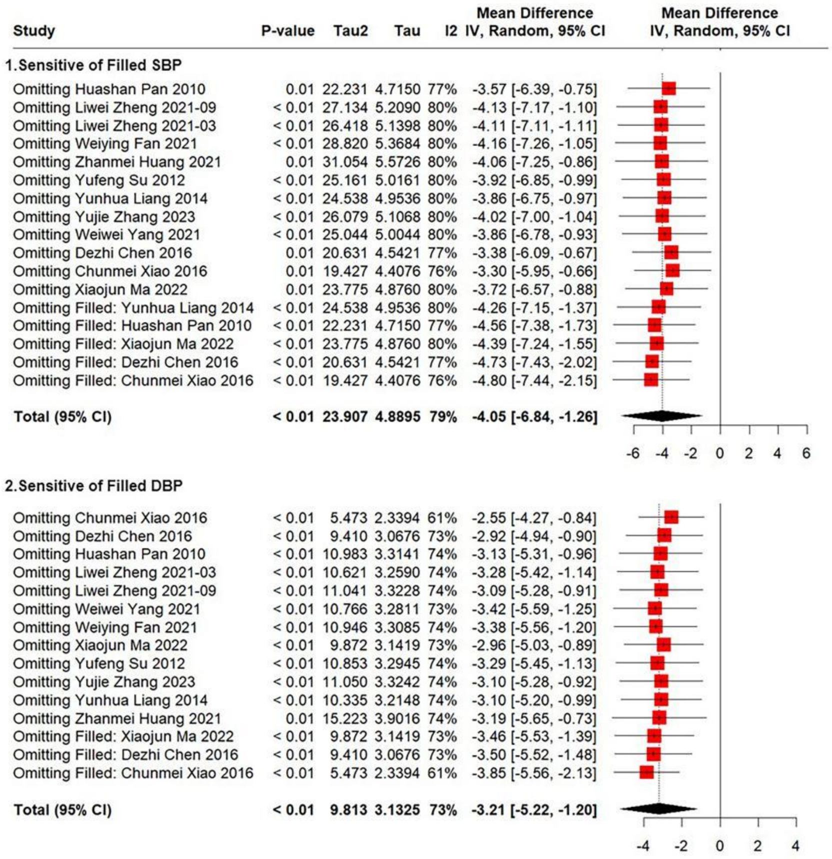

Supplementary Figure 5. Sensitive of filled SBP&DBP.

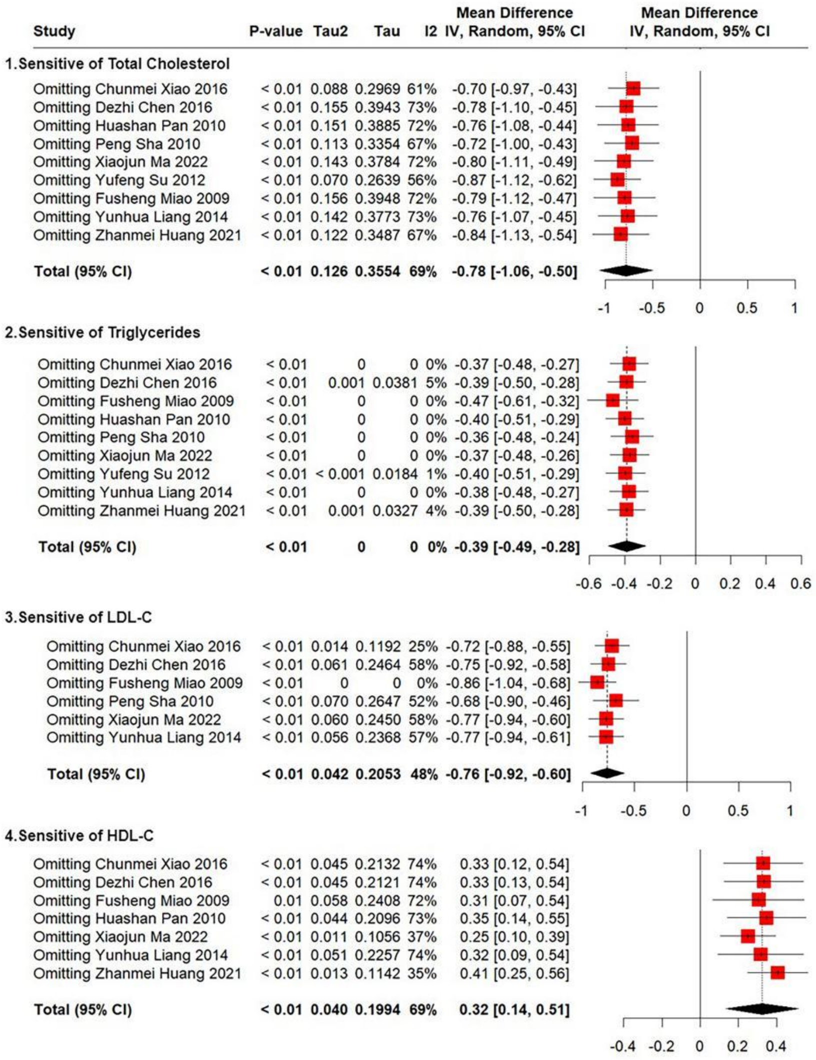

Supplementary Figure 6. Sensitive of TC & TG & LDL-C & HDL-C.

Study	P-value	Tau2	Tau	I2	Mean Difference IV, Fixed, 95% CI
**1. Sensitive of SAS**					
Omitting Weiying Fan 2021	< 0.01	5.829	2.4143	69%	-2.26 [-3.89, -0.62]
Omitting Yujie Zhang 2023	< 0.01	1.285	1.1336	74%	-3.33 [-3.81, -2.85]
Omitting Weiwei Yang 2021	< 0.01	0.294	0.5421	12%	-3.50 [-4.00, -3.01]
**Total (95% CI)**	**< 0.01**	**1.186**	**1.0892**	**61%**	**-3.37 [-3.84, -2.89]**
**2. Sensitive of PSQI**					
Omitting Liwei Zheng 2021-03	< 0.01	0.457	0.6759	39%	-2.06 [-3.23, -0.89]
Omitting Yujie Zhang 2023	< 0.01	2.402	1.5499	79%	-2.58 [-3.69, -1.46]
Omitting Zhanmei Huang 2021	< 0.01	0	0	0%	-3.46 [-4.67, -2.26]
**Total (95% CI)**	**< 0.01**	**0.999**	**0.9993**	**59%**	**-2.68 [-3.63, -1.73]**

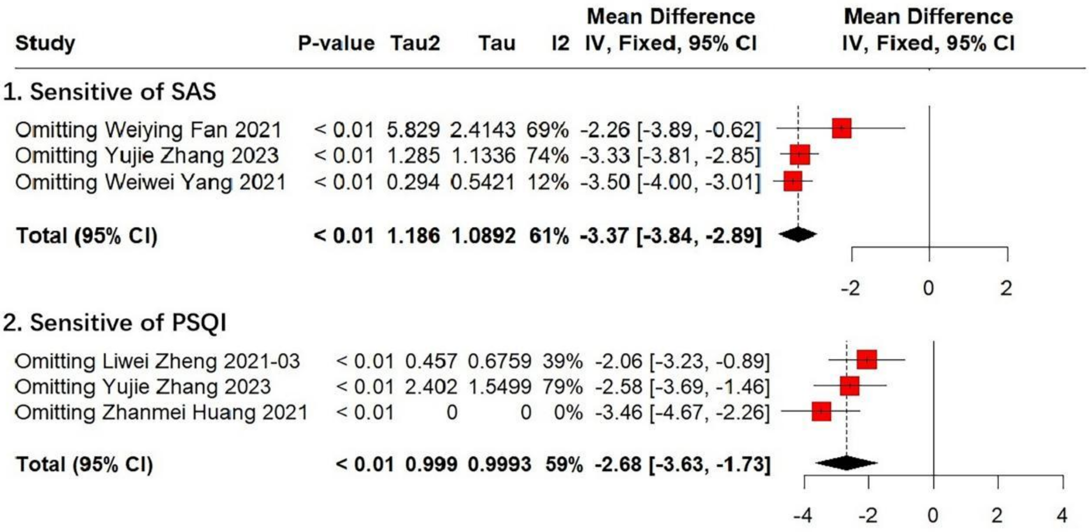

Supplementary Figure 7. Sensitive of SAS&PSQI.

1.2 Supplementary Tables

Supplementary Table 1. A detailed search strategy for PubMed.

#1	((((((((((((("Cardiovascular Diseases"[Mesh]) OR ((((((((Disease, Cardiovascular[Title/Abstract]) OR (Major Adverse Cardiac Events[Title/Abstract])) OR (Cardiac Events[Title/Abstract])) OR (Cardiac Event[Title/Abstract])) OR (Event, Cardiac[Title/Abstract])) OR (Adverse Cardiac Event[Title/Abstract])) OR (Adverse Cardiac Events[Title/Abstract])) OR (Cardiac Event, Adverse[Title/Abstract])) OR (Cardiac Events, Adverse[Title/Abstract]))) OR (Coronary[Title/Abstract])) OR ("Hypertension"[Mesh])) OR ((((Blood Pressure, High[Title/Abstract]) OR (Blood Pressures, High[Title/Abstract])) OR (High Blood Pressure[Title/Abstract])) OR (High Blood Pressures[Title/Abstract]))) OR ("Myocardial Infarction"[Mesh])) OR ("Arrhythmias, Cardiac"[Mesh])) OR (((Heart failure[Title/Abstract]) OR (Cardiac failure[Title/Abstract])) OR (Myocardial ischemia[Title/Abstract]))) OR ("Hyperlipidemias"[Mesh])) OR ("Coronary Artery Disease"[Mesh])) OR ("Stroke"[Mesh])) OR (((Apoplexy[Title/Abstract]) OR (Cerebral stroke[Title/Abstract])) OR (Cerebral infarction[Title/Abstract]))) OR ((((((Hyperlipemia[Title/Abstract])) OR(Hyperlipidemia[Title/Abstract])) OR (Lipidemia[Title/Abstract])) OR (Lipemia[Title/Abstract])) OR (Lipemias[Title/Abstract])))
#2	((("Breathing Exercises"[Mesh]) OR ("Qigong"[Mesh])) OR ((((Qi Gong[Title/Abstract]) OR (Ch'i Kung[Title/Abstract])) OR (qi gong[Title/Abstract])) OR (baduanjin[Title/Abstract]))))
#3	((("Random Allocation"[Mesh]) OR ("Cross-Over Studies"[Mesh])) OR ((((((Allocation, Random[Title/Abstract]) OR (Randomization[Title/Abstract])) OR (placebo[Title/Abstract])) OR (trial[Title/Abstract])) OR (blind[Title/Abstract])) OR (random[Title/Abstract])))
#4	#1 AND #2 AND #3
